# Advice to Consumptives

**Published:** 1869-08

**Authors:** 


### ADVICE TO CONSUMPTIVES.

*From Dixon's Scalpel.*

"A CELEBRATED impostor, whom you have appropriately designated a vulture and a jackal, professes to cure consumption by inhalation, and boasts, through the New York press, that the deaths by consumption have materially decreased in that city, since he began to minister to the consumptives. Place no confidence in his vaunted magic. Search for the true cause. Find out what hygiene has done; what a different course of treatment, generally, has effected; what honest newspapers and health magazines have done, to assist in this diminution of death from consumption. Inquire whether the increased use of exercise and good food, and the decreasing fashion of cramming the sick with medicines, have not lent their aid. Temporary relief is not a cure, though all such cases are counted cures by this unscrupulous character. The winds grow keen: do not let them drive you into the house. Dress warm. Take exercise, even at the risk of getting your nose frozen. Subscribe to some good Journal of Health, and follow its dictates, if you find them good; expose any errors in their advice, if you find them, and trust to natural remedies above all quack and patent medicines."

*From the Eclectic Medical Journal, Cincinnati, Ohio, for May.*

"The impostor referred to in the above lately made a visit to Cincinnati. He had a fine run, which lasted for several days. He succeeded in making some money, and, for a few days, in making his dupes believe that they were improving in health; but suddenly, the effects of the powerful anodynes which he used subsided, and his patients could realize their true situation. Some complained, some demanded the return of their money, others proclaimed publicly, and at the hotel, to all others who proposed to consult this celebrated impostor, that he was such. Then he received an important telegraphic dispatch, that he must return to New York. So one morning, at the usual hour for opening his office, his patients found that he had gone."

*From Hall's Journal of Health, for* 1856.

"You want air, not physic; you want pure air, not medicated air; you want nutrition, such as plenty of meat and bread will give, and they alone. Physic has no nutriment. Gaspings for air cannot cure you. Monkey capers in a gymnasium cannot cure you. Stimulants cannot cure you. If you want to get well, *go in for beef and out-door air*, and do not be deluded into the grave by advertisements and unreliable certificates."

We do not know who are the persons referred to in the papers above-named, and rather think that the "*Eclectic*" is mistaken in stating that any New Yorker has performed the part charged. It is so easy to get hold of the wrong end of a story in the papers, that we pay the slightest attention possible to such narrations; the great practical fact to which we desire to direct the special attention of the reader is, that schools of medicine so wide apart as Allopathy and Eclecticism unite so cordially in sentiment as to the only efficient means of successfully treating consumptive disease, and that their theory of to-day, is identical with our own views, as published in our JOURNAL ten years ago, and in our books, ten years before that. But the great difficulty is, not that consumption cannot be prevented, or permanently arrested, if already in progress; it is rather found in the fact, that in this fast age, men want to get well in a minute, and patronize those who most pander to their desires, and who blow their brazen trumpet with the loudest blast. Any practice that makes a man feel better soonest, is caught up with avidity and lauded to the skies, before time has been given to test the permanency of effect; so by the time the falsehood is on its feet, and often before the ink is dry which recorded it, the victim is in the grave, and can never give the contradiction.

But better, because more truthful sentiments, begin to prevail: human health is more a study; and we trust the time is not far distant, when some publication, in the nature of this Journal, will be taken by every family in the land, as ought to have been the case long, long ago.

Multitudes there are, especially of young people, who squander their money, and their more precious time, in the purchase of trashy reading, and mere animal indulgences, to end in premature death; whereas a dollar or two a year for this JOURNAL and THE SCALPEL, its profanities excepted, with a few hours a month spent in reading them, and putting their teachings in practice, would result in a healthful and genial old age.

